# Control of the Size of Silver Nanoparticles and Release of Silver in Heat Treated SiO_2_-Ag Composite Powders

**DOI:** 10.3390/ma11010080

**Published:** 2018-01-05

**Authors:** Henrika Granbohm, Juha Larismaa, Saima Ali, Leena-Sisko Johansson, Simo-Pekka Hannula

**Affiliations:** 1 Department of Chemistry and Materials Science, Aalto University School of Chemical Engineering, P.O. Box 16100, 00076 AALTO, 02150 Espoo, Finland; juha.larismaa@rockleyphotonics.com (J.L.); saima.ali@aalto.fi (S.A.); simo-pekka.hannula@aalto.fi (S.-P.H.); 2 Rockley Photonics Oy, Tietotie 3, Micronova, 02150 Espoo, Finland; 3 Department of Bioprocesses and Biosystems, Aalto University School of Chemical Engineering, P.O. Box 16300, 00076 AALTO, 02150 Espoo, Finland; leena-sisko.johansson@aalto.fi

**Keywords:** silver, silica, controlled release, particle growth, nanoparticles, nanocomposite

## Abstract

The growth of silver nanoparticles, the activation energy for silver particle growth, and the release of silver species in heat treated ${\mathrm{SiO}}_{2}$-Ag composite powders are investigated. The silver particle growth is controlled by heat treatment for 75 min of the as-synthesized ${\mathrm{SiO}}_{2}$-Ag composite powder at 300–800 °C. During heat treatment the mean size of the Ag particles increases from 10 nm up to 61 nm with increasing temperature, however, the particle size distribution widens and the mean size increases with increasing heat treatment temperature. Based on X-ray photoelectron spectroscopy (XPS) and transmission electron microscopy (TEM) studies, silver particles are crystalline and in a metallic state after annealing in all ${\mathrm{SiO}}_{2}$-Ag composite powders. The growth of Ag particles is suggested to take place via diffusion and Ostwald ripening. The activation energy for particle growth was determined as 0.14 eV. The dissolution of silver in aqueous solutions from the ${\mathrm{SiO}}_{2}$-Ag composites heat treated, at 300 °C, 600 °C, and 700 °C, was investigated by varying pH and temperature. The dissolution was reduced in all conditions with increasing silver particle size, i.e., when the total surface area of Ag particles is reduced. It is suggested that the dissolution of silver from the composite powders can conveniently be adjusted by controlling the Ag particle size by the heat treatment of the composite powder.

## 1. Introduction

Silver nanoparticles have attracted much attention during the past decade due to their versatile applications as antibacterial agents, in drug delivery, catalysis, optoelectronics, thermally conductive nanofluids and sensors [1,2,3,4,5]. Silver nanoparticles exhibit localized surface plasmon resonance (LSPR) when they are in interaction with electromagnetic radiation [2]. The unique optical properties are dependent on the size and shape of the nanoparticles [4]. Amongst other noble metals, silver has strongest surface plasmon band, giving superior performance in surface-enhanced Raman spectroscopy (SERS) sensing applications [2,6]. Silver nanoparticles have a large specific surface area, making them suitable for enhanced catalytic applications [5]. The antibacterial effects of Ag have also been known since ancient times and silver is being used in various medical devices, such as catheters [7] and in dental filling materials (glass ionomer cement) [8]. The antibacterial properties of silver are based on different forms of silver: silver, silver ions, and silver radicals [9,10]. Silver ions are prone to react with thiol and amino groups in bacterial proteins and enzymes, rendering the bacteria inactive. Free radicals of silver are reported to react with the membrane lipids, causing damage to the membrane [11]. Therefore, silver-containing materials are effective against different kinds of bacteria [12].

The Ag nanoparticles (NPs) are widely studied for antibacterial purposes. However, Ag is released from the nanoparticles by oxidation [13]. The release kinetics are affected by many parameters, such as Ag concentration, Ag surface functionalization and temperature [13,14,15]. The composition of the medium for dissolution also influences the release of ${\mathrm{Ag}}^{+}$. For instance, the ${\mathrm{Ag}}^{+}$ release diminishes when dissolved oxygen is removed from water [14]. However, uncontrolled release of silver can cause cytotoxicity in cells [16,17,18]. The cytotoxicity is also shown to be size dependent [18], and smaller sizes (<10 nm) have been shown to be more efficient [19]. Therefore, different stabilizers, such as citrate [20,21,22,23] or polyvinylpyrrolidone (PVP) [13,24,25,26,27] are utilized to control the size of silver and the release of silver into the surrounding media. Different kinds of trigger release mechanisms, such as photoactivation and pH-triggers have been studied, to control the release of silver, e.g., to a specific place or for prolonged times [28,29,30].

The silver NPs are generally synthesized as hybrid organic or inorganic metallic composites to overcome the agglomeration of silver nanoparticles, as they tend to agglomerate due to higher surface energy and van der Waals forces [2,4]. However, the organic binders such as polymers and ligands attenuate the optical properties due to surface encapsulation [4,5], in addition to their non-biocompatibility in some cases [2]. Silver-silica composites have gained more attention amongst the inorganic hybrids due to their stability, chemical inertness and transparency in UV and IR regions [4,29]. The surface can also be functionalized for the biocompatible applications [4].

Generally, the reported synthesis of silver silica composites involves a multistep process that requires a long reaction time. Different coupling reagents such as polyvinylpyrrolidone (PVP), 3-aminopropyltrimethoxysilane (APS) and polyoxyethylene(5)nonylphenyl ether are used for the synthesis [4]. In the present work, we report one step synthesis of ${\mathrm{SiO}}_{2}$-Ag composite powder without any reducing or coupling agents. The growth of silver nanoparticles in ${\mathrm{SiO}}_{2}$-Ag composite powders are examined after heat treatment for 75 min between 300 °C and 800 °C, in order to show that the size of the Ag NPs and their effective surface area in the synthesized composite powders can be controlled by subsequent heat treatment. The activation energy of Ag particle growth is determined. Furthermore, the release of silver in water is probed for the ${\mathrm{SiO}}_{2}$-Ag composite powders heat treated at 300 °C, 600 °C and 700 °C under different pH and temperature. Up until today, there are no systematic studies on the release of silver species from a silica particle matrix with no clear porous structure, as reported herein. The controlled release of silver from the present composites can be utilized e.g., in antimicrobial wound dressings [12].

## 2. Results and Discussion

### 2.1. Morphology and Structure

**Figure 1:**
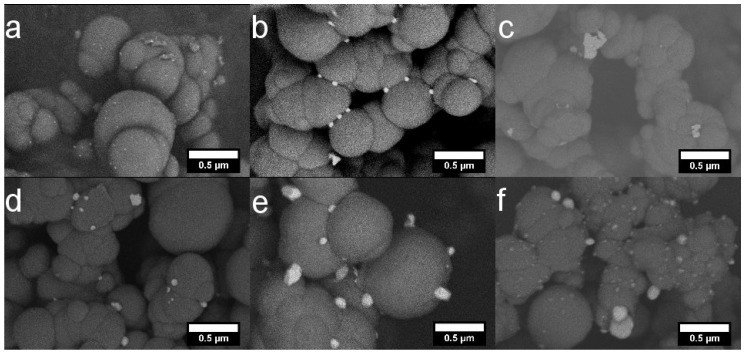
scanning electron microscopy (SEM) images of ${\mathrm{SiO}}_{2}$-Ag powders heat treated at (**a**) 300 °C; (**b**) 400 °C; (**c**) 500 °C; (**d**) 600 °C; (**e**) 700 °C and (**f**) 800 °C for 75 min. All scale bars are 0.5 μm.

**Figure A1:**
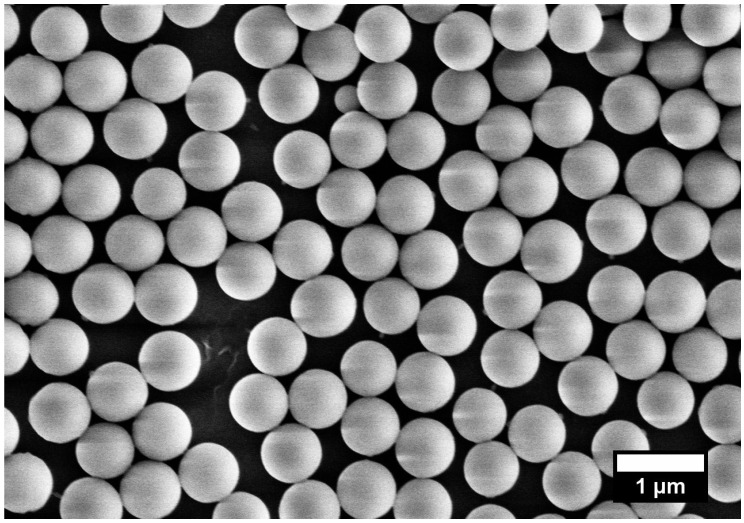
SEM image of ${\mathrm{SiO}}_{2}$ powder.

The size of the ${\mathrm{SiO}}_{2}$ particles in the ${\mathrm{SiO}}_{2}$ powder was determined by measuring the diameter of fifty ${\mathrm{SiO}}_{2}$ particles from a scanning electron microscopy (SEM) image. An SEM image of the pure ${\mathrm{SiO}}_{2}$ powder sample with a mean size of 670 nm is shown in Figure A1. SEM images of all heat treated ${\mathrm{SiO}}_{2}$-Ag composite powders at the same magnification are shown in Figure 1. The Ag particles appear as light spots and silica in grey. The Ag particles are observed on top of the ${\mathrm{SiO}}_{2}$ matrix. The modified Stöber process and the subsequent heat treatment produces irregularly shaped ${\mathrm{SiO}}_{2}$-Ag clusters in various sizes, contrary to the reference ${\mathrm{SiO}}_{2}$ particles, which are spherical without agglomeration. The reaction mechanism for silver nitrate in the water-ethanol-ammonia solution is described in Equations (1)–(3) [31]. ${\mathrm{AgNO}}_{3}$ reacts with ${\mathrm{NH}}_{4}\mathrm{OH}$ forming the unstable AgOH (Equation (1)). AgOH then reacts further to form ${\mathrm{Ag}}_{2}O$ (Equation (2)). ${\mathrm{Ag}}_{2}O$ dissolves in the excess ${\mathrm{NH}}_{4}\mathrm{OH}$ and reacts forming the $[\mathrm{Ag}{\left({\mathrm{NH}}_{3}\right)}_{2}]\mathrm{OH}$ complex (Equation (3)). The $[\mathrm{Ag}{\left({\mathrm{NH}}_{3}\right)}_{2}]\mathrm{OH}$ complex is the source for the silver particles throughout the silica matrix. The as-synthesized ${\mathrm{SiO}}_{2}$-Ag composite powder is white in color, however, the color of the powder changes to brownish-yellow upon heat treatment at 300–800 °C, indicating growth of silver particles.

(1) $$AgNO_{3}+NH_{4}OH⟶AgOH↓+NH_{4}NO_{3}$$

(2) $$AgOH⟶Ag_{2}O↓+H_{2}O$$

(3) $$Ag_{2}O↓+H_{2}O+NH_{4}OH⟶\left[Ag{\left(NH_{3}\right)}_{2}\right]OH+H_{2}O$$

**Figure 2:**
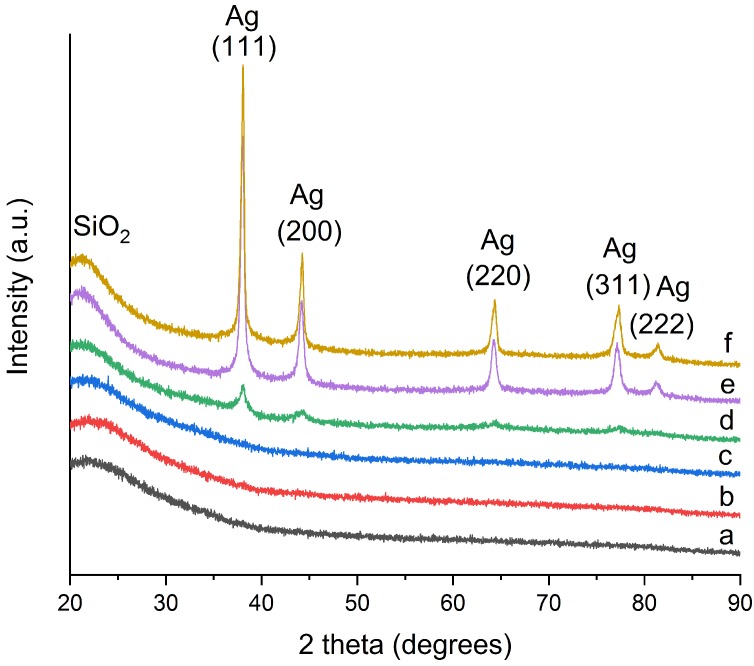
X-ray diffraction patterns of (**a**) the dried ${\mathrm{SiO}}_{2}$-Ag powder and ${\mathrm{SiO}}_{2}$-Ag powders heat treated at (**b**) 300 °C; (**c**) 400 °C; (**d**) 500 °C; (**e**) 700 °C and (**f**) 800 °C for 75 min.

Figure 2 shows the X-ray diffraction patterns of the heat treated ${\mathrm{SiO}}_{2}$-Ag powders. The hump at about 2θ 22° is attributed to the amorphous ${\mathrm{SiO}}_{2}$ matrix. The amorphous hump is sharpened upon the increased heat treatment temperature and the peak position moves slightly to smaller 2θ values, suggesting gradual ordering in the amorphous ${\mathrm{SiO}}_{2}$ structure. No silver peaks are detected after heat treatment below 600 °C, however, at or above 600 °C the silver peaks appear. The silver peaks (2θ) are found at 37.9°, 44.1°, 64.2°, 77.1° and 81.2°, corresponding to (111), (200), (220), (311) and (222) crystalline planes (pattern No. 01-087-0597). The absence of diffraction peaks for silver at lower heat treatment temperatures is attributed to the small size of silver, which is consistent with previous research [32]. The silver peaks become more prominent with increasing temperature, indicating increased silver particle size.

**Figure 3:**
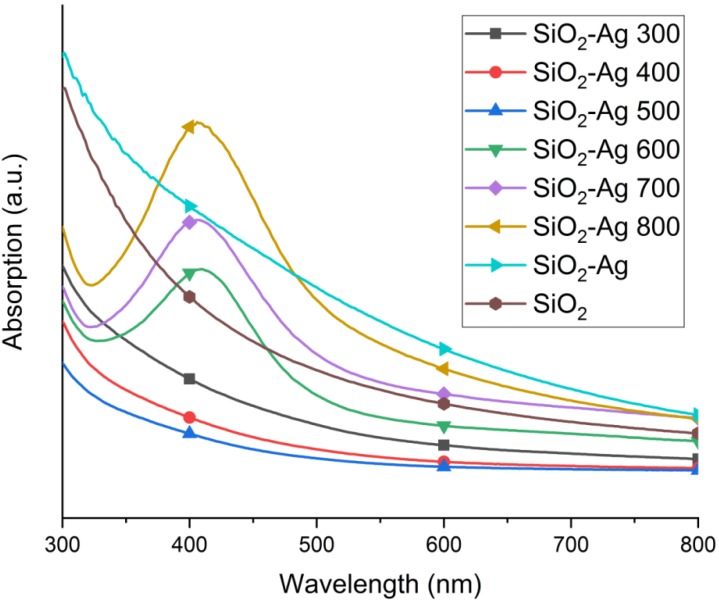
UV-vis spectra of ${\mathrm{SiO}}_{2}$ and ${\mathrm{SiO}}_{2}$-Ag powders, showing the silver SPR peak around 410 nm.

**Figure A2:**
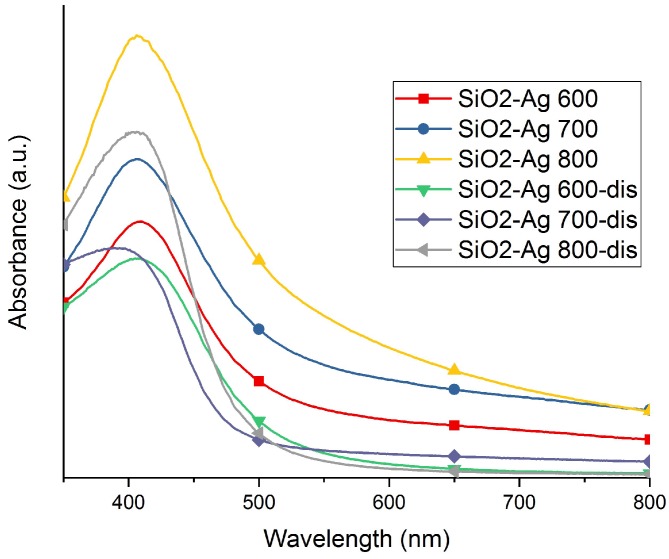
UV-vis spectra of ${\mathrm{SiO}}_{2}$-Ag 600, ${\mathrm{SiO}}_{2}$-Ag 700 and ${\mathrm{SiO}}_{2}$-Ag 800 with Ag dissolved by ${\mathrm{HNO}}_{3}$ from the surface and ${\mathrm{SiO}}_{2}$-Ag 600, ${\mathrm{SiO}}_{2}$-Ag 700 and ${\mathrm{SiO}}_{2}$-Ag 800 powders showing the Ag plasmon peak for reference.

Silver can also be observed by probing the surface plasmon resonance (SPR) peak around 410 nm by UV-vis spectroscopy. The optical absorption spectra of the powders are shown in Figure 3. The intensity of the broad absorption band starting at 300 nm decreases with heat treatment temperature. The silver plasmon peak is visible in ${\mathrm{SiO}}_{2}$-Ag powder samples heat treated at 600 °C, 700 °C and 800 °C, which is consistent with the XRD results (Figure 2). The silver plasmon peak position blue-shifts slightly from 410 nm for ${\mathrm{SiO}}_{2}$-Ag 600 to 406 nm for both ${\mathrm{SiO}}_{2}$-Ag 700 and ${\mathrm{SiO}}_{2}$-Ag 800. The plasmon peak intensity increases with increasing average size of the Ag NPs. The plasmon peak is sustained due to Ag NPs within ${\mathrm{SiO}}_{2}$, even when the silver particles from the surface are dissolved (Supporting information, Figure A2). The plasmon peak positions are slightly blue shifted; ${\mathrm{SiO}}_{2}$-Ag 600 from 410 to 407 nm, ${\mathrm{SiO}}_{2}$-Ag 700 from 406 nm to 390 nm, and ${\mathrm{SiO}}_{2}$-Ag 800 from 406 nm to 403 nm. The ${\mathrm{SiO}}_{2}$-Ag 700 sample displayed a blue shift of 16 nm, suggesting that smaller sized Ag particles are present after dissolving Ag particles from the surface than before dissolution [33]. If the spherical shape of the Ag NPs is retained, this means that it takes longer for the Ag particles inside the silica matrix to grow compared to those on the surface. This is justified by the probable differences in diffusion rate of Ag in the bulk and the surface as well as the higher constrains of particle growth in the bulk, as opposed to on the free surface. On the other hand, it is known that the thickness of the silica on the silver particles also affects the plasmon peak position due to scattering [33,34,35].

**Figure 4:**
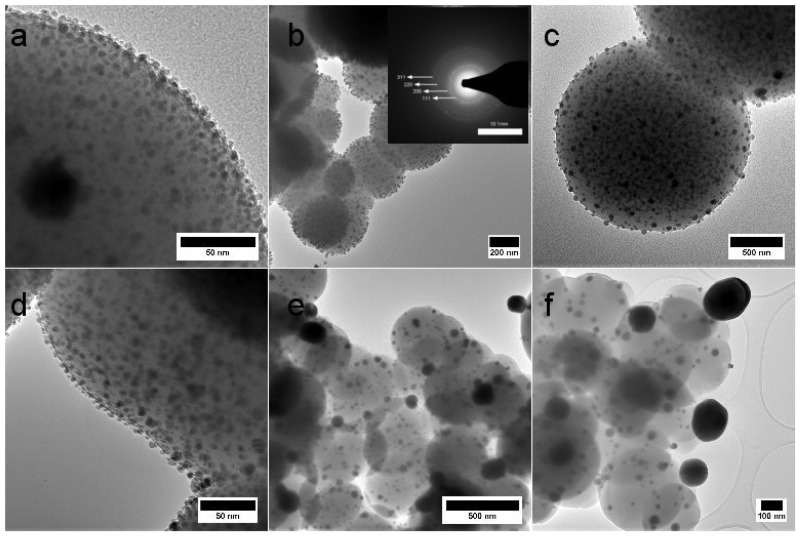
TEM images of (**a**) the dried ${\mathrm{SiO}}_{2}$-Ag powder and the powders heat treated at (**b**) 300 °C; (**c**) 400 °C; (**d**) 500 °C; (**e**) 700 °C and (**f**) 800 °C for 75 min. The lighter parts represent the silica matrix, whereas the darker spots are silver particles. The insert in (**b**) displays the electron diffraction pattern revealing crystalline silver particles after heat treatment at 300 °C. Scale bars in (**a**) 50 nm; (**b**) 200 nm; (**c**) 500 nm; (**d**) 50 nm; (**e**) 500 nm and (**f**) 100 nm.

The growth of Ag NPs and the detailed representation of the ${\mathrm{SiO}}_{2}$-Ag materials are depicted in the transmission electron microscopy (TEM) images in Figure 4. The Ag NPs appear as darker spots in various sizes in and on the ${\mathrm{SiO}}_{2}$ matrix. The electron diffraction pattern in the insert of Figure 4b illustrates the crystalline nature of silver particles heat treated at 300 °C. The growth of the Ag particles is studied by measuring the Ag NP size distribution from several TEM and SEM micrographs and is given in Table 1. The size of Ag particle is represented by its diameter. Altogether, 60–495 Ag particles per sample were measured. There is a clear growth in mean Ag NP size after heat treatment, however, small Ag NPs are present after all heat treatment temperatures. They are evenly distributed on the surface of the ${\mathrm{SiO}}_{2}$ particle surface up to 600 °C. At 700 °C and 800 °C, the Ag particles have grown into larger clusters and have started to desquamate from the surface. Nevertheless, small Ag NPs are still present in the samples.

**Figure 5:**
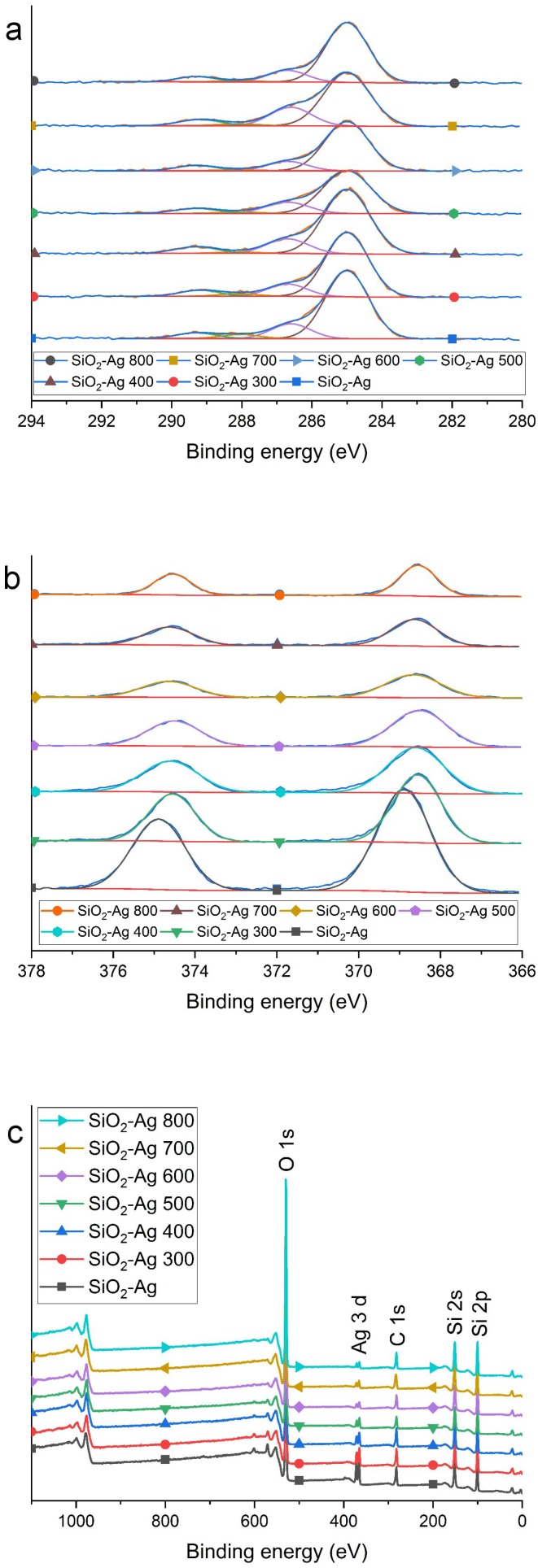
High resolution XPS spectra showing (**a**) the C 1 s high resolution spectra and (**b**) the Ag 3d peaks for the powders. XPS survey spectra of (**c**) all ${\mathrm{SiO}}_{2}$-Ag powders.

According to X-ray photoelectron spectroscopy (XPS) analysis, the composite powder surfaces consisted of silica and silver only, see Figure 5. The only significant changes observed at the surface were related to silver. Firstly, the measured amount of silver at the surface was 1.8 at. % in the non-heat treated reference, and it decreased monotonously from 1.3 at. % to 0.4 at. % when heat treatment temperature rose from 300 °C to 800 °C. Increase in the Ag particle size due to heat treatments, combined with the XPS analysis depth of only a few nanometers explains this result, which is in good accordance with the observed Ag particle sizes in TEM. As for the surface chemistry, we found no signs of silver oxidation, but in the non-heat treated reference the Ag $3d_{5/2}$ signal was observed at 268.9 eV, while it appeared at a slightly lower binding energy, 268.5 eV, in all heat treated samples, see Figure 5b. A similar shift has been reported on some silver alloys [36,37] and on some organic complexes containing oxygen, so we believe that the higher binding energy in the non-heat treated sample is due to the interaction between colloidal silver and the ${\mathrm{SiO}}_{2}$ matrix.

**Figure A3:**
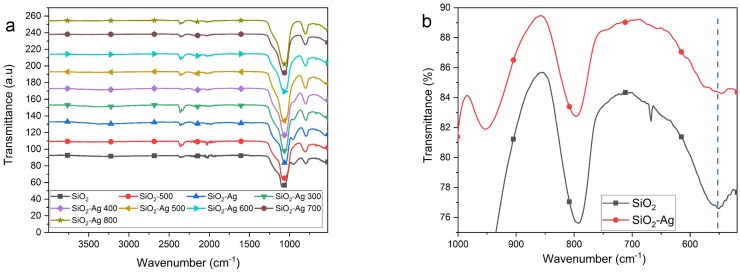
FTIR spectra of (**a**) all ${\mathrm{SiO}}_{2}$-Ag composite samples, ${\mathrm{SiO}}_{2}$ and a ${\mathrm{SiO}}_{2}$ sample annealed at 500°C. A close up of the Si–O–Ag interaction at 554 ${\mathrm{cm}}^{-1}$ of (**b**) the as-prepared ${\mathrm{SiO}}_{2}$-Ag and ${\mathrm{SiO}}_{2}$ for comparison.

The interaction between the Ag NPs and the ${\mathrm{SiO}}_{2}$ matrix and functional groups in the composite samples were investigated by Fourier Transform Infrared spectroscopy (FTIR), as observed in Figure A3. The two strong bands between 1000–1200 ${\mathrm{cm}}^{-1}$ and 750–850 ${\mathrm{cm}}^{-1}$ are ascribed to the asymmetric and symmetric Si–O–Si stretching vibrations, respectively [38,39,40]. The band around 950 ${\mathrm{cm}}^{-1}$ originates from the Si–OH bonds [39,40]. The peaks get weaker upon heat treatment till 400 °C and the peak disappeared for the samples heat treated at 500 °C, indicating –OH group removal with heat treatment. Figure A3b shows a small band around 554 ${\mathrm{cm}}^{-1}$ ascribed to the Si–O–Ag linkages [40]. The band appears in the ${\mathrm{SiO}}_{2}$-Ag composites suggesting bonding between the Ag NPs and the oxygen bonded to silicon, which supports the interpretation of the XPS spectra.

The surface area of the ${\mathrm{SiO}}_{2}$-Ag powders was studied by BET analysis (Table 1). A specific surface area of 5.2 $m^{2}\;g^{-1}$ was obtained for a pure ${\mathrm{SiO}}_{2}$ sample prepared without the addition of ${\mathrm{AgNO}}_{3}$. The results show that the specific surface area of the ${\mathrm{SiO}}_{2}$-Ag powders decrease when the heat treatment temperature is increased, with the exception of the ${\mathrm{SiO}}_{2}$-Ag 800 powder. The surface area decrease from 5.2 $m^{2}\;g^{-1}$ for ${\mathrm{SiO}}_{2}$ to smaller values for the composite powders may be due to agglomeration of the ${\mathrm{SiO}}_{2}$ matrix. The change in specific surface area is investigated after the powders were ${\mathrm{HNO}}_{3}$ treated to remove the Ag on the surface of the composites. The surface area decreases for all ${\mathrm{SiO}}_{2}$-Ag samples, except for the as-prepared ${\mathrm{SiO}}_{2}$-Ag powder and ${\mathrm{SiO}}_{2}$-Ag 700 powder. The decrease in specific surface area is probably due to the removal of Ag NPs from the surface, together with possible densification and sintering of the powders, however, it should be noted that the accuracy of BET measurements are typically ±5%. The slight increase in the specific surface area of the two samples after dissolution may result from the surface area of dents remaining after removal of Ag NPs from the surface. The largest difference in surface area is found for the ${\mathrm{SiO}}_{2}$-Ag 300 powder, as the removal of the smallest Ag particles from the ${\mathrm{SiO}}_{2}$ surface provides the largest surface area.

The formation and growth of particles can be divided into three stages: nucleation phase, diffusion-based growth and Ostwald ripening [41,42]. Diffusion-based growth takes place at large supersaturation values, whereas Ostwald ripening proceeds when smaller particles dissolve to form larger particles [42]. The nucleation step occurs during the synthesis or drying step of the as-prepared ${\mathrm{SiO}}_{2}$-Ag sample, considering the as-prepared powder contains Ag particles after drying as confirmed by XPS and TEM. The Ag particle size (diameter) increases during heat treatment of the ${\mathrm{SiO}}_{2}$-Ag powder in air.

**Figure 6:**
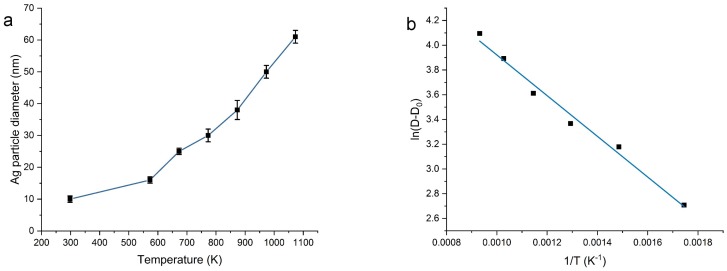
Graphical representation of the mean diameter growth of Ag particles in different temperatures (**a**). The error bars represent the standard error. An Arhennius type plot based on Equation (6) for determination of activation energy ($Q_{A}$) for Ag NP growth on the ${\mathrm{SiO}}_{2}$ matrix (**b**).

Let us assume that the growth of Ag NPs follows a typical kinetic law of type

(4) $$\frac{dD}{dt}=kt^{n}$$

where *D* is the Ag particle size, *t* is time, and *k* and *n* are constants at constant temperature. The temperature dependence of the growth is hidden in term *k*. If the temperature dependence follows an Arrhenius type of relation, we can write the equation as:

(5) $$k=Ae^{\frac{-Q_{A}}{k_{B}T}}$$

where $k_{B}$ is the Boltzmann constant, $Q_{A}$ the activation energy, *T* is the temperature and *A* is a constant. By insertion of (5) into (4), integrating and rearranging, we obtain the following equation, assuming a constant heat treatment time:

(6) $$ln\left(D-D_{0}\right)=\frac{-Q_{A}}{k_{B}T}+A^{t}$$

where *D* is the particle size, $D_{0}$ is the particle size at *t* = 0, and $A^{t}$ is a constant. The activation energy $Q_{A}$ is obtained from the slope of the graph in Figure 6b, i.e., by plotting $ln(D-D_{0})$ as a function of 1/*T*.

Based on the linear fit in Figure 6b, an activation energy of 0.14 eV is obtained for the Ag NP growth. Activation energies for Ag particle nucleation and growth in silica implanted at room temperature with energetic Ag ions have previously been found as 0.064 eV below 800 °C and 0.40 eV above 800 °C [43]. The study was carried out by Ag ion-implantation into silica glass substrates, where subsequent heat treatment leads to diffusion of the implanted atoms, nucleation and growth of Ag crystallites. Other studies [44,45,46,47,48] have found significantly higher activation energies of 1.8–5.2 eV for Ag particle growth in Ag-doped ${\mathrm{SiO}}_{2}$ films on soda-lime glass. However, there are substantial differences between these studies: (i) the nucleation and growth of Ag are investigated in ${\mathrm{SiO}}_{2}$ films, i.e., matrix affects the nucleation [48]; (ii) some studies [48] deal with narrow temperature ranges (e.g., 570–600 °C); (iii) holding times are varying; and (iv) materials are made with different preparation methods.

Furthermore, Ag is prone to oxidation. In another report, oxidation of Ag was described during heat treatment in air in a 55 nm ${\mathrm{SiO}}_{2}$ nanofilm [42]. The oxidation occurred by oxygen uptake by the silica nanofilm restricting Ag growth. However, oxidation of Ag was prevented by shortening the heat treatment times down to 10 min. and keeping the heat treatment temperature above 550 °C [42,48]. The ${\mathrm{SiO}}_{2}$ matrix seems to protect Ag from oxidation at lower temperatures even for the heat treatment time of 75 min. used in this work, as the XPS results shown in Figure 5 affirm.

During Ostwald ripening, particles grow larger in size due to the dissolution of smaller particles. The Ostwald ripening stage can be distinguished in TEM images, which display larger Ag particles with a low concentration of small particles. A close study of Figure 4 and other TEM images not shown here, suggests that a large number of smaller Ag particles are sustained up to 600 °C, but are more scarce in ${\mathrm{SiO}}_{2}$-Ag 700 and ${\mathrm{SiO}}_{2}$-Ag 800. However, in each case, there are still small Ag particles present. Ostwald ripening can also be detected spectroscopically by observing the SPR peak of Ag. The Ag particle size increase should present higher SPR peak intensities, whereas a decrease in particle concentration should lower the SPR peak intensity. The SPR peak in Figure 3 provides this information, as the concentration of powder to solvent is kept constant at 1 $\mathrm{mg}\cdot {\mathrm{mL}}^{-1}$. The Ag SPR peak intensity increases as the temperature is increased from 600 °C to 800 °C, suggesting the presence of larger particles at elevated temperatures. The increase in SPR peak intensity thus supports the conclusion that Ostwald ripening takes place during heat treatment. Additional experiments are needed in order to identify detailed growth mechanisms and the role of different forms of diffusion in the process.

### 2.2. Release of Silver

The release of silver (${\mathrm{Ag}}^{0}$ and ${\mathrm{Ag}}^{+}$) from the ${\mathrm{SiO}}_{2}$-Ag 300, ${\mathrm{SiO}}_{2}$-Ag 600 and ${\mathrm{SiO}}_{2}$-Ag 700 powder samples were examined to obtain the concentration of silver from the surface of the ${\mathrm{SiO}}_{2}$ matrix. Control samples were measured to obtain a maximum silver concentration leached from the ${\mathrm{SiO}}_{2}$-Ag 300, ${\mathrm{SiO}}_{2}$-Ag 600 and ${\mathrm{SiO}}_{2}$-Ag 700 powder samples. The theoretical maximum for the control samples with 3.3 $\mathrm{mg}\cdot L^{-1}$ ${\mathrm{SiO}}_{2}$-Ag loading was 285 $\mathrm{mg}\cdot L^{-1}$ Ag, corresponding to 431 $\mathrm{mg}\cdot L^{-1}$ for a 5.0 $\mathrm{mg}\cdot L^{-1}$ ${\mathrm{SiO}}_{2}$-Ag loading. The obtained release from the control samples with 3.3 $\mathrm{mg}\cdot L^{-1}$ ${\mathrm{SiO}}_{2}$-Ag loading was 212 $\mathrm{mg}\cdot L^{-1}$, 19.1 $\mathrm{mg}\cdot L^{-1}$, and 192 $\mathrm{mg}\cdot L^{-1}$ from the ${\mathrm{SiO}}_{2}$-Ag 300, ${\mathrm{SiO}}_{2}$-Ag 600 and ${\mathrm{SiO}}_{2}$-Ag 700 samples, respectively. The calculated values were 321 $\mathrm{mg}\cdot L^{-1}$, 29 $\mathrm{mg}\cdot L^{-1}$, 290 $\mathrm{mg}\cdot L^{-1}$ for the ${\mathrm{SiO}}_{2}$-Ag 300, ${\mathrm{SiO}}_{2}$-Ag 600 and ${\mathrm{SiO}}_{2}$-Ag 700 samples, respectively, for a 5.0 $\mathrm{mg}\cdot L^{-1}$ ${\mathrm{SiO}}_{2}$-Ag loading. The silver concentration was unexpectedly ca 10 times lower for the ${\mathrm{SiO}}_{2}$-Ag 600 control sample than ${\mathrm{SiO}}_{2}$-Ag 300 and ${\mathrm{SiO}}_{2}$-Ag 700 control samples. Comparing the obtained release and the theoretical release of the control samples, there is an indication that the silver species in ${\mathrm{SiO}}_{2}$-Ag 300 and ${\mathrm{SiO}}_{2}$-Ag 700 is mostly on the surface since up to 74% of silver was released during acid digestion. However, the case of ${\mathrm{SiO}}_{2}$-Ag 600 is a bit peculiar with a release of only 6.7% of the theoretical maximum. The control samples were prepared in triplicate, so it is unlikely to be a systematic error. This would suggest that the silver has diffused into the silica matrix, as previously observed by Babapour et al. in the case of Ag in a ${\mathrm{SiO}}_{2}$ film [49].

**Figure 7:**
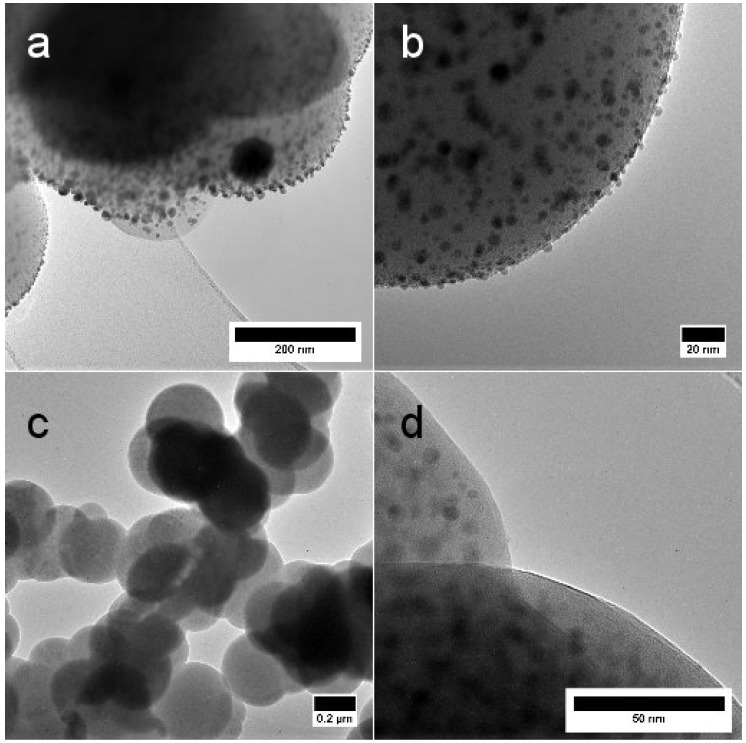
TEM images of ${\mathrm{SiO}}_{2}$-Ag 600 powder (**a**,**b**) and the Ag dissolved from the surface by ${\mathrm{HNO}}_{3}$ from the ${\mathrm{SiO}}_{2}$-Ag 600 powder. The lighter parts represent the silica matrix, whereas the darker spots are silver particles. Scale bars in (**a**) 200 nm; (**b**) 20 nm; (**c**) 0.2 μm and (**d**) 50 nm.

The ${\mathrm{SiO}}_{2}$-Ag 600 was imaged by TEM after ${\mathrm{HNO}}_{3}$ digestion as shown in Figure 7. Figure 7a,b show the ${\mathrm{SiO}}_{2}$-Ag 600 sample before acid digestion and Ag particles are observed both on the surface and inside the silica matrix. Ag is dissolved from the surface after acid digestion in Figure 7c,d, however, Ag particles are still observed inside the silica matrix. Hence, only a fraction of the theoretical maximum of Ag in the sample is on the surface of the silica matrix.

**Figure 8:**
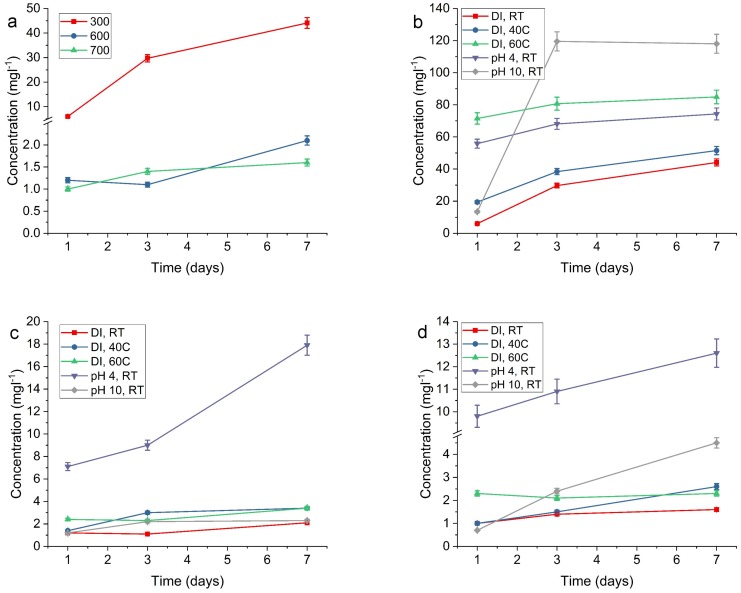
The release of silver from (**a**) ${\mathrm{SiO}}_{2}$-Ag 300, ${\mathrm{SiO}}_{2}$-Ag 600 and ${\mathrm{SiO}}_{2}$-Ag 700 during one to seven days in de-ionized water at room temperature. Comparison of Ag release at room temperature, 40 °C, 60 °C, pH 4, and pH 10 for (**b**) ${\mathrm{SiO}}_{2}$-Ag 300; (**c**) ${\mathrm{SiO}}_{2}$-Ag 600; and (**d**) ${\mathrm{SiO}}_{2}$-Ag 700. The time scale is in days. In the legend: DI is de-ionized water, RT is room temperature.

**Figure A4:**
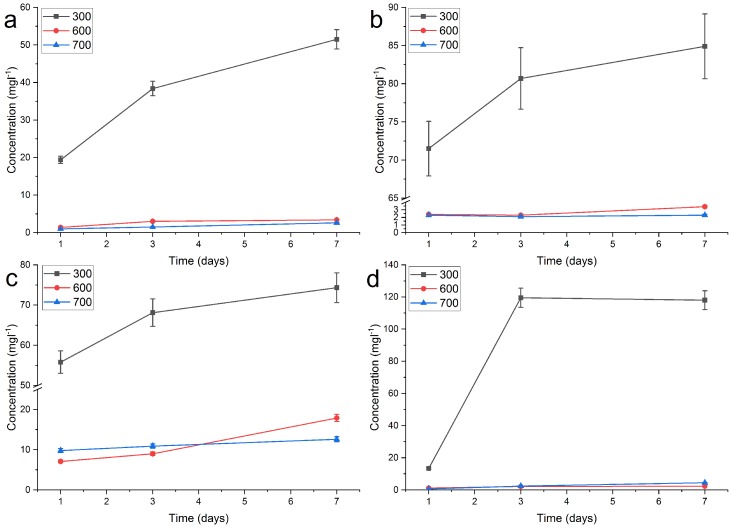
Comparison of Ag release at (**a**) 40 °C; (**b**) 60 °C; (**c**) pH 4; and (**d**) pH 10 for ${\mathrm{SiO}}_{2}$-Ag 300, ${\mathrm{SiO}}_{2}$-Ag 600, and ${\mathrm{SiO}}_{2}$-Ag 700, marked as 300, 600 and 700 in the graphs, respectively. The time scale is in days.

The first leaching test was performed in de-ionized water at room temperature for one, three and seven days, which is marked in Figure 8 as DI, RT. The results are depicted in Figure 8 and Figure A4, and listed in Table A1. Figure 8a shows the highest release of silver species for the ${\mathrm{SiO}}_{2}$-Ag 300 sample. The ${\mathrm{SiO}}_{2}$-Ag 300 shows a release of 6.0 $\mathrm{mg}\cdot L^{-1}$ already after one day, increasing to 44.1 $\mathrm{mg}\cdot L^{-1}$ for dissolution at seven days. The corresponding silver concentrations for the ${\mathrm{SiO}}_{2}$-Ag 600 sample is from 1.2 $\mathrm{mg}\cdot L^{-1}$ to 2.1 $\mathrm{mg}\cdot L^{-1}$ and for the ${\mathrm{SiO}}_{2}$-Ag 700 sample from 1.0 $\mathrm{mg}\cdot L^{-1}$ to 1.6 $\mathrm{mg}\cdot L^{-1}$ for 1 day to seven days dissolutions. This means that the ${\mathrm{SiO}}_{2}$-Ag 300 displays from six times up to 27 times larger silver release as compared to the ${\mathrm{SiO}}_{2}$-Ag 700 sample. The ${\mathrm{SiO}}_{2}$-Ag 300 sample has the smallest average silver particle size of the studied ${\mathrm{SiO}}_{2}$-Ag samples on the ${\mathrm{SiO}}_{2}$ surface, indicating that smaller Ag NPs (10 nm) in the ${\mathrm{SiO}}_{2}$-Ag 300 dissolve or desquamate readily from the composite compared to larger NPs in ${\mathrm{SiO}}_{2}$-Ag 600 and ${\mathrm{SiO}}_{2}$-Ag 700.

The release of Ag from ${\mathrm{SiO}}_{2}$-Ag was also investigated at elevated temperatures of 40 °C and 60 °C. The ${\mathrm{SiO}}_{2}$-Ag 300 and ${\mathrm{SiO}}_{2}$-Ag 600 composites displayed a trend of increasing Ag concentration with longer dissolution time. The concentration of Ag in the ${\mathrm{SiO}}_{2}$-Ag 300 sample increased with increasing temperature. The ${\mathrm{SiO}}_{2}$-Ag 600 sample released similar amounts of Ag in both 40 °C and 60 °C. The Ag concentration increased at 40 °C for the ${\mathrm{SiO}}_{2}$-Ag 700 sample, however, at 60 °C the concentration is unchanged. The temperature change appears to have a larger effect on smaller Ag particles leading them to readily dissolve, whereas a temperature change does not have the same effect for ${\mathrm{SiO}}_{2}$-Ag 600 and ${\mathrm{SiO}}_{2}$-Ag 700 samples.

The Ag concentration is expected to be larger at pH 4, as Ag dissolves in acidic environments. All samples displayed increasing Ag release with increased dissolution time, ${\mathrm{SiO}}_{2}$-Ag 300 having the most extensive release of Ag with 74.3 $\mathrm{mg}\cdot L^{-1}$ at seven days. The ${\mathrm{SiO}}_{2}$-Ag 600 sample measured 17.9 $\mathrm{mg}\cdot L^{-1}$ and ${\mathrm{SiO}}_{2}$-Ag 700 12.6 $\mathrm{mg}\cdot L^{-1}$ at seven days, which correlates with the increasing Ag size. At pH 10, ${\mathrm{SiO}}_{2}$-Ag 300 displayed the largest Ag release of 28% of the theoretical maximum release of Ag and 38% of the Ag concentration in the control sample. The ${\mathrm{SiO}}_{2}$-Ag 600 and ${\mathrm{SiO}}_{2}$-Ag 700 samples did not follow this trend, i.e., they exhibited the largest release at pH 4.

## 3. Materials and Methods

### 3.1. Preparation of $SiO_{2}$-Ag Composites

The ${\mathrm{SiO}}_{2}$-Ag composite powders were prepared by a modified Stöber method [50]. In brief, ${\mathrm{AgNO}}_{3}$ (≥99.0%, Sigma-Aldrich, St. Louis, MO, USA) powder was dissolved in a solution of $H_{2}O$, ${\mathrm{NH}}_{4}\mathrm{OH}$ (25%, JT Baker, Center Valley, PA, USA), and ethanol (96.1 vol %, Altia, Helsinki, Finland) under magnetic stirring. Subsequently, tetraethyl orthosilicate (TEOS, ≥99.0%, Sigma-Aldrich) was slowly added and stirred for 2 h. The molar ratio of the precursors was 19.0:1.0:1487.8:220.5:471.4 TEOS:${\mathrm{AgNO}}_{3}$:${\mathrm{CH}}_{3}{\mathrm{CH}}_{2}\mathrm{OH}$:${\mathrm{NH}}_{4}\mathrm{OH}$:$H_{2}O$. The product was then centrifuged and dried at room temperature. The dried product was heat treated in air at temperatures between 300 and 800 °C for 75 min. The dried powder was white in color and turned into deeper brownish-yellow color with increasing heat treatment temperature. A sample of ${\mathrm{SiO}}_{2}$ powder was prepared with the same recipe excluding the ${\mathrm{AgNO}}_{3}$ addition. The powder samples are named according to the heat treatment temperature, e.g., “${\mathrm{SiO}}_{2}$-Ag 300” is heat treated at 300 °C for 75 min.

### 3.2. Dissolution of Silver

Tests for the dissolution of silver from the ${\mathrm{SiO}}_{2}$-Ag composite powders were performed on ${\mathrm{SiO}}_{2}$-Ag 300, ${\mathrm{SiO}}_{2}$-Ag 600 and ${\mathrm{SiO}}_{2}$-Ag 700 samples with a sample loading of 5.0 $g\cdot L^{-1}$. The release studies were made in aqueous solutions. All tests were made in triplicates for one, three and seven days. Test temperatures were room temperature, 40 °C and 60 °C. The media was de-ionized water and de-ionized water with the pH adjusted to 4 with ${\mathrm{HNO}}_{3}$ or to 10 with NaOH. The mixtures were then filtered through 0.45 μm nylon syringe filters, acidified with ${\mathrm{HNO}}_{3}$ and silver concentration analyzed. The control samples (sample loading of 3.3 $g\cdot L^{-1}$) were prepared by dissolving the silver from the ${\mathrm{SiO}}_{2}$-Ag samples in concentrated ${\mathrm{HNO}}_{3}$.

### 3.3. Characterization

The powders were characterized utilizing X-ray Diffraction (XRD), which was carried out on a PANalytical X’pert Powder Pro diffractometer (PANalytical, EA Almelo, The Netherlands) using Cu κ($\alpha_{1/2}$) λ($\alpha_{1}$) = 1.54060 Å radiation in the range of 20–90° (2θ). Hitachi S-4700 cold field emission gun scanning electron microscope (FEG-SEM, Tokyo, Japan) was used to study particle sizes, structure and appearance of the powders, while a Tecnai F20 G2 200 kV FEG (Tecnai, Hillsboro, OR, USA) transmission electron microscope (TEM) was used to analyze Ag particle size, morphology and the composition of selected powders. The Ag particle sizes were determined by measuring the diameter of the Ag particles with the ImageJ measurement tool from a number of TEM and SEM images. The TEM samples were prepared by dispersing the ${\mathrm{SiO}}_{2}$-Ag powder sample in ethanol by ultrasonication for 3 min and placing a drop of the dispersion on the TEM grid. The UV-vis absorbance spectra for the silver plasmon peak measurements were carried out on a Hitachi U-5100 UV-vis spectrophotometer. Surface chemical compositions of composite powders were determined with X-ray photoelectron spectroscopy (XPS), using AXIS Ultra electron spectrometer by Kratos Analytical (Manchester, UK) and 285.0 eV as the reference value for the CC component in C 1 s [51]. The FTIR measurements were carried out on a Nicolet 380 FT-IR (Thermo Fisher Scientific, Waltham, MA, USA). The specific surface area and porosity of the ${\mathrm{SiO}}_{2}$-Ag samples were examined by nitrogen adsorption–desorption isotherms at 77 K using a TriStar II 3020 equipment and the ${\mathrm{SiO}}_{2}$ were analyzed using Bel Belmaster Mini II equipment (BEL, Tuggerah, Australia). Brunauer–Emmet–Teller (BET) analysis was used for the specific surface area. The silver concentrations were determined by inductively coupled plasma atomic emission spectroscopy (ICP-AES) utilizing a Perkin Elmer ICP-AES Optima 7100 DV (Perkin Elmer, Waltham, MA, USA), the error for the measurements were estimated as ±5 %. The silver emission spectrum was probed at 328.068 nm and 338.289 nm.

## 4. Conclusions

The Ag NP size in ${\mathrm{SiO}}_{2}$-Ag composite powders was successfully controlled by heat treatment for 75 min at temperatures between 300 °C and 800 °C. The silver is shown to be in metallic form without utilization of reducing agent before heat treatment. The activation energy for growth of Ag particles was determined to be 0.14 eV, which is well below the activation energy for Ag NP growth in ${\mathrm{SiO}}_{2}$ films. The release of Ag species from three samples was investigated; ${\mathrm{SiO}}_{2}$-Ag 300, ${\mathrm{SiO}}_{2}$-Ag 600 and ${\mathrm{SiO}}_{2}$-Ag 700 in de-ionized water at room temperature, at 40 °C and 60 °C, and at pH 4 and pH 10 for one to seven days time. The silver concentration increased or plateaued after three days, depending on the release conditions. The results suggest that the silver release can be controlled by varying the loading of the powders and dissolution conditions. The Ag release was clearly Ag size (surface area) dependent.

## Figures and Tables

**Table 1:** Statistics of silver nanoparticle sizes, amount of measured particles (N total) and their standard error (SE). The specific surface area for the ${\mathrm{SiO}}_{2}$-Ag powders heat treated at different temperatures (measurement accuracy ±5%). The specific surface area determined by nitrogen adsorption-desorption is also presented for the ${\mathrm{SiO}}_{2}$-Ag samples with the surface Ag dissolved by ${\mathrm{HNO}}_{3}$ (marked with “-dis”).

T (°C)	N Total Particles	Mean ± SE (nm)	Min (nm)	Max (nm)	Surface Area ($m^{2}\;g^{-1}$)	Surface Area-Dis ($m^{2}\;g^{-1}$)
RT	60	10 ± 1	4	48	4.2	4.5
300	175	16 ± 1	4	65	5.2	4.2
400	186	25 ± 1	4	122	4.9	4.3
500	247	30 ± 2	4	199	4.9	4.6
600	188	28 ± 3	4	210	4.7	4.3
700	298	50 ± 2	7	202	4.6	4.8
800	495	61 ± 2	4	283	5.2	4.6

## Abbreviations

The following abbreviations are used in this manuscript:

APS	3-aminopropyltrimethoxysilane
BET	Brunauer-Emmmet-Teller
DI	De-ionized water
FEG	Field Emission Gun
FTIR	Fourier Transform Infrared
ICP-AES	Inductively Coupled Plasma Atomic Emission Spectroscopy
NP	Nanoparticle
PVP	Polyvinylpyrrolidone
RT	Room temperature
SD	Standard Deviation
SEM	Scanning Electron Microscopy
SPR	Surface Plasmon Resonance
TEM	Transmission Electron Microscopy
TEOS	Tetraethyl orthosilicate
XRD	X-Ray Diffraction
XPS	X-ray Photoelectron Spectroscopy

## Appendix A. Supporting Information

**Table A1:** Silver release ($\mathrm{mg}\cdot L^{-1}$) from ${\mathrm{SiO}}_{2}$-Ag 300, ${\mathrm{SiO}}_{2}$-Ag 600 and ${\mathrm{SiO}}_{2}$-Ag 700 for 1, 3 and 7 days at room temperature (RT) in de-ionized water (DI), at 40 °C and 60 °C, and pH 4 and pH 10. The  errors are estimated as ±5%.

Sample	${\mathrm{SiO}}_{2}$-Ag 300			${\mathrm{SiO}}_{2}$-Ag 600			${\mathrm{SiO}}_{2}$-Ag 700		
Days	1	3	7	1	3	7	1	3	7
RT, DI	6.0	29.7	44.1	1.2	1.1	2.1	1.0	1.4	1.6
40 °C, DI	19.4	38.4	51.5	1.4	3.0	3.4	1.0	1.5	2.6
60 °C, DI	71.5	80.7	84.9	2.4	2.3	3.4	2.3	2.1	2.3
pH 4, RT	55.8	68.1	74.3	7.1	9.0	17.9	9.8	10.9	12.6
pH 10, RT	13.4	119.5	118.0	1.2	2.2	2.3	0.7	2.4	4.5

## References

[1] Tzounis L., Logothetidis S. (2017). Fe_3_O_4_@SiO_2_ core shell particles as platforms for the decoration of Ag nanoparticles. Mater. Today-Proc..

[2] Tzounis L., Contreras-Caceres R., Schellkopf L., Jehnichen D., Fischer D., Cai C., Uhlmann P., Stamm M. (2014). Controlled growth of Ag nanoparticles decorated onto the surface of SiO_2_ spheres: A nanohybrid system with combined SERS and catalytic properties. RSC Adv..

[3] Niitsoo O., Couzis A. (2011). Facile synthesis of silver core – silica shell composite nanoparticles. J. Colloid Interf. Sci..

[4] Bahadur N.M., Furusawa T., Sato M., Kurayama F., Siddiquey I.A., Suzuki N. (2011). Fast and facile synthesis of silica coated silver nanoparticles by microwave irradiation. J. Colloid Interf. Sci..

[5] Cai Y., Tan F., Qiao X., Wang W., Chen J., Qiu X. (2016). Room-temperature synthesis of silica supported silver nanoparticles in basic ethanol solution and their antibacterial activity. RSC Adv..

[6] Ju J., Liu W., Perlaki C., Chen K., Feng C., Liu Q. (2017). Sustained and Cost Effective Silver Substrate for Surface Enhanced Raman Spectroscopy Based Biosensing. Sci. Rep..

[7] Furno F., Morley K.S., Wong B., Sharp B.L., Arnold P.L., Howdle S.M., Bayston R., Brown P.D., Winship P.D., Reid H.J. (2004). Silver nanoparticles and polymeric medical devices: A new approach to prevention of infection?. J. Antimicrob. Chemother..

[8] Coughlan A., Boyd D., Douglas C.W.I., Towler M.R. (2008). Antibacterial coatings for medical devices based on glass polyalkenoate cement chemistry. J. Mater. Sci.-Mater. M.

[9] Chernousova S., Epple M. (2013). Silver as antibacterial agent: Ion, nanoparticle, and metal. Angew. Chem. Int. Ed..

[10] Prabhu S., Poulose E.K. (2012). Silver nanoparticles: Mechanism of antimicrobial action, synthesis, medical applications, and toxicity effects. Int. Nano Lett..

[11] Kim J., Kuk E., Yu K., Kim J.H., Park S., Lee H., Kim S., Park Y., Park Y., Hwang C.Y. (2007). Antimicrobial effects of silver nanoparticles. Nanomedicine.

[12] Mosselhy D., Granbohm H., Hynönen U., Ge Y., Palva A., Nordström K., Hannula S.P. (2017). Nanosilver–silica composite: Prolonged antibacterial effects and bacterial interaction mechanisms for wound dressings. Nanomaterials (Basel).

[13] Kittler S., Greulich C., Diendorf J., Köller M., Epple M. (2010). Toxicity of Silver Nanoparticles Increases during Storage Because of Slow Dissolution under Release of Silver Ions. Chem. Mater..

[14] Liu J., Hurt R.H. (2010). Ion Release Kinetics and Particle Persistence in Aqueous Nano-Silver Colloids. Environ. Sci. Technol..

[15] Liu J., Sonshine D.A., Shervani S., Hurt R.H. (2010). Controlled Release of Biologically Active Silver from Nanosilver Surfaces. ACS Nano.

[16] Gaiser B.K., Fernandes T.F., Jepson M.A., Lead J.R., Tyler C.R., Baalousha M., Biswas A., Britton G.J., Cole P.A., Johnston B.D. (2012). Interspecies comparisons on the uptake and toxicity of silver and cerium dioxide nanoparticles. Environ. Toxicol. Chem..

[17] Miura N., Shinohara Y. (2009). Cytotoxic effect and apoptosis induction by silver nanoparticles in HeLa cells. Biochem. Bioph. Res. Commun..

[18] Gliga A.R., Skoglund S., Odnevall Wallinder I., Fadeel B., Karlsson H.L. (2014). Size-dependent cytotoxicity of silver nanoparticles in human lung cells: The role of cellular uptake, agglomeration and Ag release. Part. Fibre Toxicol..

[19] Agnihotri S., Mukherji S., Mukherji S. (2014). Size-controlled silver nanoparticles synthesized over the range 5–100 nm using the same protocol and their antibacterial efficacy. RSC Adv..

[20] Bastus N.G., Merkoci F., Piella J., Puntes V. (2014). Synthesis of Highly Monodisperse Citrate-Stabilized Silver Nanoparticles of up to 200 nm: Kinetic Control and Catalytic Properties. Chem. Mater..

[21] He D., Bligh M.W., Waite T.D. (2013). Effects of Aggregate Structure on the Dissolution Kinetics of Citrate-Stabilized Silver Nanoparticles. Environ. Sci. Technol.

[22] Tejamaya M., Römer I., Merrifield R.C., Lead J.R. (2012). Stability of Citrate, PVP, and PEG Coated Silver Nanoparticles in Ecotoxicology Media. Environ. Sci. Technol..

[23] Henglein A., Giersig M. (1999). Formation of Colloidal Silver Nanoparticles: Capping Action of Citrate. J. Phys. Chem. B.

[24] Romih T., Jemec A., Kos M., Hocevar S.B., Kralj S., Makovec D., Drobne D. (2016). The role of PVP in the bioavailability of Ag from the PVP-stabilized Ag nanoparticle suspension. Environ. Pollut..

[25] Cho K.H., Park J.E., Osaka T., Park S.G. (2005). The study of antimicrobial activity and preservative effects of nanosilver ingredient. Electrochim. Acta.

[26] Yin B., Ma H., Wang S., Chen S. (2003). Electrochemical Synthesis of Silver Nanoparticles under Protection of Poly(*N*-vinylpyrrolidone). J. Phys. Chem. B.

[27] He B., Tan J.J., Liew K.Y., Liu H. (2004). Synthesis of size controlled Ag nanoparticles. J. Mol. Catal. A- Chem..

[28] Elashnikov R., Lyutakov O., Ulbrich P., Svorcik V. (2016). Light-activated polymethylmethacrylate nanofibers with antibacterial activity. Mat. Sci. Eng. C.

[29] Fang W., Yang J., Gong J., Zheng N. (2012). Photo- and pH-Triggered Release of Anticancer Drugs from Mesoporous Silica-Coated Pd@Ag Nanoparticles. Adv. Funct. Mater..

[30] Lyutakov O., Goncharova I., Rimpelova S., Kolarova K., Svanda J., Svorcik V. (2015). Silver release and antimicrobial properties of PMMA films doped with silver ions, nano-particles and complexes. Mat. Sci. Eng. C.

[31] Turova N. (2011). Inorganic Chemistry in Tables.

[32] Wang J.X., Wen L.X., Wang Z.H., Chen J.F. (2006). Immobilization of silver on hollow silica nanospheres and nanotubes and their antibacterial effects. Mater. Chem. Phys..

[33] Rainville L., Dorais M.C., Boudreau D. (2013). Controlled synthesis of low polydispersity Ag@SiO_2_ core-shell nanoparticles for use in plasmonic applications. RSC Adv..

[34] Amendola V., Bakr O.M., Stellacci F. (2010). A Study of the Surface Plasmon Resonance of Silver Nanoparticles by the Discrete Dipole Approximation Method: Effect of Shape, Size, Structure, and Assembly. Plasmonics.

[35] De G., Licciulli A., Massaro C., Tapfer L., Catalano M., Battaglin G., Meneghini C., Mazzoldi P. (1996). Silver nanocrystals in silica by sol-gel processing. J. Non-Cryst. Solids.

[36] Grünert W., Schlögl R., Karge H. (1993). Investigations of zeolites by photoelectron and ion scattering spectroscopy. 1. New applications of surface spectroscopic methods to zeolites by a high-temperature measurement technique. J. Phys. Chem..

[37] Rodriguez J., Hrbek J. (1994). Synergistic interactions in trimetallic bonding: A comparison of the ZnNM Ru(001) (NM = Cu, Ag or Au) systems. Surf. Sci..

[38] Ramalingam S., Devi L.B., Raghava Rao J., Unni Nair B. (2014). Rapid hydrogenation: Perfect quasi architecture (Ag@SiO_2_NPs) as a substrate for nitrophenol reduction. RSC Adv..

[39] Peszke J., Dulski M., Nowak A., Balin K., Zubko M., Sulowicz S., Nowak B., Piotrowska-Seget Z., Talik E., Wojtyniak M. (2017). Unique properties of silver and copper silica-based nanocomposites as antimicrobial agents. RSC Adv..

[40] Racles C., Nistor A., Cazacu M. (2013). A silica-silver nanocomposite obtained by sol-gel method in the presence of silver nanoparticles. Cent. Eur. J. Chem..

[41] Ekimov A. (1996). Growth and optical properties of semiconductor nanocrystals in a glass matrix. J. Lumin..

[42] Jiménez J., Sendova M., Hartsfield T., Sendova-Vassileva M. (2011). In situ optical microspectroscopy of the growth and oxidation of silver nanoparticles in silica thin films on soda-lime glass. Mater. Res. Bull..

[43] Ila D., Baglin J., Zimmerman R. (2015). Nano-Crystal Formation and Growth from High-Fluence Ion Implantation of Au, Ag or Cu in Silica. Phys. Proc..

[44] Jimenez J., Sendova M. (2011). In situ optical microspectroscopy approach for the study of metal transport in dielectrics via temperature- and time-dependent plasmonics: Ag nanoparticles in SiO_2_ films. J. Chem. Phys..

[45] Jimenez J., Sendova M., Liu H. (2011). Evolution of the optical properties of a silver-doped phosphate glass during thermal treatment. J. Lumin..

[46] Jimenez J., Sendova M. (2011). Diffusion activation energy of Ag in nanocomposite glasses determined by in situ monitoring of plasmon resonance evolution. Chem. Phys. Lett..

[47] Jimenez J., Sendova M. (2012). Kinetics of Ag nanoparticle growth in thick SiO_2_ films: An in situ optical assessment of Ostwald ripening. Mater. Chem. Phys..

[48] Jiménez J.A., Sendova M. (2013). Unfolding diffusion-based Ag nanoparticle growth in SiO_2_ nanofilms heat-treated in air via in situ optical microspectroscopy. Opt. Mater..

[49] Babapour A., Akhavan O., Azimirad R., Moshfegh A. (2006). Physical characteristics of heat-treated nano-silvers dispersed in sol-gel silica matrix. Nanotechnology.

[50] Larismaa J., Honkanen T., Ge Y., Söderberg O., Friman M., Hannula S.P. (2011). Effect of annealing on Ag-doped submicron silica powder prepared with modified Stöber Method. Mater. Sci. Forum.

[51] Beamson G., Briggs D. (1993). High Resolution XPS of Organic Polymers—The Scienta ESCA300 Database.

