# Correction: Granbohm, H, et al. Control of the Size of Silver Nanoparticles and Release of Silver in Heat Treated SiO_2_-Ag Composite Powders. *Materials* 2018, *11*, 80

**DOI:** 10.3390/ma11091617

**Published:** 2018-09-05

**Authors:** Henrika Granbohm, Juha Larismaa, Saima Ali, Leena-Sisko Johansson, Simo-Pekka Hannula

**Affiliations:** 1 Department of Chemistry and Materials Science, Aalto University School of Chemical Engineering, P.O. Box 16100, 00076 AALTO, 02150 Espoo, Finland; juha.larismaa@rockleyphotonics.com (J.L.); saima.ali@aalto.fi (S.A.); simo-pekka.hannula@aalto.fi (S.-P.H.); 2 Rockley Photonics Oy, Tietotie 3, Micronova, 02150 Espoo, Finland; 3 Department of Bioprocesses and Biosystems, Aalto University School of Chemical Engineering, P.O. Box 16300, 00076 AALTO, 02150 Espoo, Finland; leena-sisko.johansson@aalto.fi

In the published article “Control of the Size of Silver Nanoparticles and Release of Silver in Heat Treated SiO_2_-Ag Composite Powders” [1] a reference was omitted in the caption of Figure 4b. A citation to [2] (Reference [12] in the reference list of [1]) has been added. This does not change the numbering of the reference list in [1].

The difference between the silver particle sizes reported in [1,2] for the SiO_2_–Ag sample annealed at 300 °C results from the different measuring techniques used in the two papers. As stated in [2], the silver size was solely determined from TEM images resulting in average particle size of 5 nm ± 2 nm, while in [1] both SEM and TEM images were used resulting in average particle size of 16 nm, the standard deviation for the latter being ± 10 nm. With TEM, the silver particles can be imaged and counted only if the thickness of silver particles together with silica is small enough to be electron transparent. On the other hand, SEM images take into account all sizes of particles, and reveal larger particles found close to particle–particle interfaces (Figure 1 of [1]) and not visible in the TEM images. Taking these silver particles into account broadens the size distribution and increases the average size.

We also found an error in the caption for Figure 2 of [1]. The caption should read as follows: “X-ray diffraction patterns of SiO_2_–Ag powders heat treated at (a) 300 °C; (b) 400 °C; (c) 500 °C; (d) 600 °C; (e) 700 °C; and (f) 800 °C for 75 min”.

The changes do not affect the results. We apologize for the inconvenience this has caused and we would like to thank the editorial office for notifying us about the omitted citation. The manuscript will be updated and the original will remain online on the article webpage, with a reference to this Correction.
